# A unique swim bladder-inner ear connection in a teleost fish revealed by a combined high-resolution microtomographic and three-dimensional histological study

**DOI:** 10.1186/1741-7007-11-75

**Published:** 2013-07-04

**Authors:** Tanja Schulz-Mirbach, Martin Heß, Brian D Metscher, Friedrich Ladich

**Affiliations:** 1 Department of Behavioural Biology, University of Vienna, Althanstrasse 14, 1090 Vienna, Austria; 2 Department of Biology II, Zoology, Ludwig-Maximilians-University, Großhadernerstrasse 2, 82152 Martinsried, Germany; 3 Department of Theoretical Biology, University of Vienna, Althanstrasse 14, 1090 Vienna, Austria

**Keywords:** Cichlidae, Otolithic end organs, Accessory auditory structures, High-resolution microCT, Histological serial sectioning, Lagena

## Abstract

**Background:**

In most modern bony fishes (teleosts) hearing improvement is often correlated with a close morphological relationship between the swim bladder or other gas-filled cavities and the saccule or more rarely with the utricle. A connection of an accessory hearing structure to the third end organ, the lagena, has not yet been reported. A recent study in the Asian cichlid *Etroplus maculatus* provided the first evidence that a swim bladder may come close to the lagena. Our study was designed to uncover the swim bladder-inner ear relationship in this species. We used a new approach by applying a combination of two high-resolution techniques, namely microtomographic (microCT) imaging and histological serial semithin sectioning, providing the basis for subsequent three-dimensional reconstructions. Prior to the morphological study, we additionally measured auditory evoked potentials at four frequencies (0.5, 1, 2, 3 kHz) to test the hearing abilities of the fish.

**Results:**

*E. maculatus* revealed a complex swim bladder-inner ear connection in which a bipartite swim bladder extension contacts the upper as well as the lower parts of each inner ear, a condition not observed in any other teleost species studied so far. The gas-filled part of the extension is connected to the lagena via a thin bony lamella and is firmly attached to this bony lamella with connective material. The second part of the extension, a pad-like structure, approaches the posterior and horizontal semicircular canals and a recessus located posterior to the utricle.

**Conclusions:**

Our study is the first detailed report of a link between the swim bladder and the lagena in a teleost species. We suggest that the lagena has an auditory function in this species because the most intimate contact exists between the swim bladder and this end organ. The specialized attachment of the saccule to the cranial bone and the close proximity of the swim bladder extension to the recessus located posterior to the utricle indicate that the saccule and the utricle also receive parallel inputs from the swim bladder extension. We further showed that a combination of non-destructive microCT imaging with histological analyses on the same specimen provides a powerful tool to decipher and interpret fine structures and to compensate for methodological artifacts.

## Background

The inner ear in fishes comprises the three semicircular canals and the three otolithic end organs, namely the utricle, the saccule and the lagena [1,2]. In modern bony fishes (teleosts) the sensory epithelium of each otolithic end organ is overlain by a single calcium carbonate concretion, the otolith [3]. Movement of the fish in a sound field leads to the lagged movement of the denser otolith relative to the fish’s body and the sensory epithelium, which stimulates the sensory hair cells [3,4]. Fishes using solely this stimulation pathway have limited auditory abilities [4-6]. To improve hearing (that is, to expand frequency detection up to several thousand Hertz and/or to increase sensitivities), species across different taxonomic groups have evolved accessory auditory structures. Inner ears in these species are close to or connected with either (1) intracranial gas cavities (anabantoids, mormyrids) or (2) anterior extensions of the swim bladder (for example, some holocentrids, sciaenids, cichlids, clupeids) or (3) are linked to the swim bladder via a chain of ossicles and ligaments (Weberian apparatus of the otophysans) [7,8]. The gas bladders, oscillating in a sound field, then transmit energy to the inner ear endolymph, which again results in movement of the otolith relative to the sensory epithelium.

In fishes, it is still difficult to assign a certain vestibular or auditory function to one of the end organs [4]. Although it is clear that the semicircular canals in the inner ear detect only angular accelerations, it is assumed that utricle, saccule, and lagena may serve in both vestibular and auditory functions [4,9]. It is thus likely that all three otolithic end organs are involved in hearing to different degrees in different species [10]. Nevertheless, physiological and anatomical data indicate that the saccule and, to a yet unknown extent, the lagena are the main auditory end organs. Extirpation experiments by von Frisch and his colleagues demonstrated in Eurasian minnows *Phoxinus phoxinus* (cypriniforms) that removal of the utricle and semicircular canals did not affect hearing at all, whereas simultaneous removal of the saccule and lagena completely eliminated the detection of sound frequencies above 130 Hz [11]. Dijkgraaf [12] demonstrated that bilateral extirpation of the saccule and the lagena in the black goby *Gobius niger* decreased their auditory sensitivity and eliminated sound detection above 400 Hz. The complete removal of both inner ears led to a further decrease in the detectable frequency range. Schuijf [13] showed that cutting the nerve root of the saccule and lagena of one inner ear in the cod *Gadus morhua* reduced the ability to localize a sound source. In addition, in most species possessing accessory auditory structures — otophysans (cypriniforms, siluriforms, characiforms and gymnotiforms), anabantoids, mormyrids, and some holocentrids — the swim bladder or gas-filled cavities contact the saccule (for a review see [8]). None of the so far investigated teleost species, however, possesses a connection between the swim bladder and the lagena.

Ramcharitar *et al*. [14] described swim bladder extensions in the silver perch *Bairdiella chrysoura* that came close to the lagena without, however, directly contacting it or any of the other two end organs. Recently, Schulz-Mirbach *et al*. [15] provided the first evidence, based on microtomographic (microCT) scans and dissections, that the swim bladder extensions in the Asian cichlid species *Etroplus maculatus* approach the lagena; they further showed that *E. maculatus* has improved hearing abilities compared to other cichlid species lacking such a swim bladder-inner ear connection. From that study, it was not completely clear whether the swim bladder directly contacted the lagena. Moreover, those findings partly contradicted data published by Dehadrai [16], who illustrated and described the swim bladder-inner ear relationship in *Etroplus suratensis* without showing or mentioning the lagena. Sparks [17] noted intracranial bullae of the swim bladder contacting the inner ears, which was not found in other studies [15,16]. These former studies [15-17] did not use the high-resolutions of microCT imaging or histological analysis we used in our study. Although Dehadrai [16] performed histological analyses of the swim bladder, he did not describe or illustrate histological sections showing the contact region of swim bladder extensions and inner ears. Nonetheless, histological analysis is necessary in order to determine whether the swim bladder extension abuts the lagena, abuts the bone surrounding the lagena, or is firmly attached to the lagena or to the bone via connective tissue in *E. maculatus*. Several of the steps during sample preparation for the histological analysis (decalcification, dehydration, embedding, and sectioning procedures), however, may cause artifacts such as shrinkage, distortion or loss of important sections [18]. We, therefore, applied a new methodological approach, a combination of two high-resolution techniques: (1) non-destructive high-resolution microCT imaging by which the fixed and stained sample was investigated prior to decalcification, dehydration, and embedding; and (2) histological serial sectioning of the same specimen. This approach aimed to identify potential artifacts during histological sample preparation. Prior to these morphological investigations, we additionally tested hearing abilities using the auditory evoked potential (AEP) recording technique [19,20]. In our study, we, therefore, asked (1) whether a direct anatomical connection exists between the swim bladder extensions and inner ears in *E. maculatus* and, if so, (2) whether the extension is linked only to the lagena or also to the saccule and (3) whether internal bullae are present.

We found a close anatomical link between swim bladder and lagena via a thin bony lamella, indicating that the lagena in *E. maculatus* plays a role in audition. A saccule attachment to the neurocranium and the close vicinity between the swim bladder extension and the posterior recessus of the upper inner ear also suggest an involvement of the utricle and, thus, of all three end organs in improved auditory sensitivities.

## Results

### Auditory measurements

Specimens of *E. maculatus* revealed highest auditory sensitivity at 0.5 kHz and a decrease at higher frequencies (Table 1). Thresholds measured at the four tested frequencies (0.5 to 3 kHz) were similar to those found in larger individuals investigated in a previous study [15] (Table 1).

**Table 1:** **Mean auditory sensitivities (± s.e.m.) of *E. maculatus *determined in the present and in the prior study by Schulz-Mirbach *et al*.**[15]

	**Present study**	**Schulz-Mirbach *et al*. [**[15]**]**
Mean SL (mm)	28 ± 0.9	37 ± 0.9
Number of specimens	4	8
Frequency (kHz)	Sound pressure level (dB re 1 μPa)	
0.5	72 ± 1.5	70 ± 0.8
1	78 ± 2.6	80 ± 0.2
2	108 ± 1.6	109 ± 1.2
3	112 ± 1.3	114 ± 1.0

*SL* standard length.

### Swim bladder-inner ear connection

**Figure 1:**
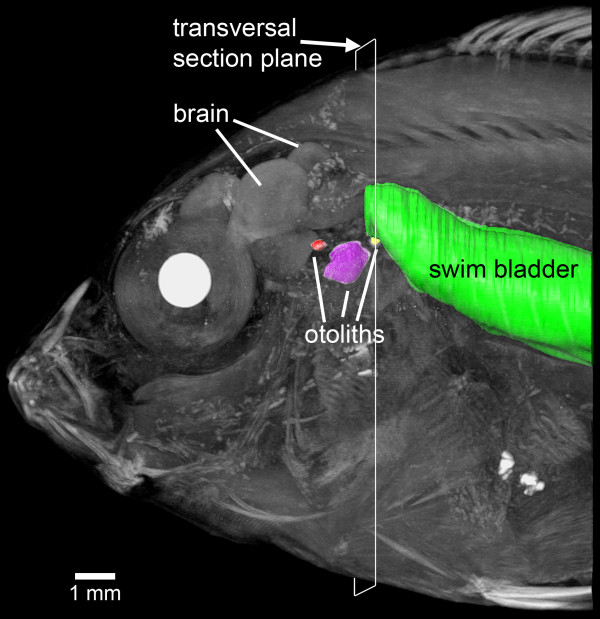
**Overview of the swim bladder-inner ear relationship in *E. maculatus*.** Left lateral view of the anterior body (volume rendering based on microCT data) with surface renderings of the swim bladder (green) and the utricular (red), saccular (purple) and lagenar (yellow) otoliths, superimposed. Reconstructions are based on low-resolution microCT imaging using the SkyScan 1174 (voxel size 36 μm isotropic). The white frame indicates the section plane of transversal sections shown in Figure 2. Scale bar, 1 mm. microCT, microtomography.

**Figure 2:**
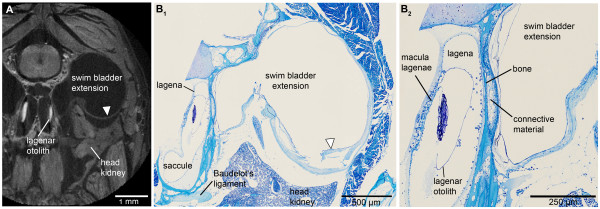
**Transversal sections of the head of *E. maculatus *(SL = 29 mm) displaying the swim bladder-inner ear connection.** Sections are based on **(A)** high-resolution microCT imaging and **(B)** histological serial sections. **(A)** represents a slightly oblique slice through the microCT data stack, largely adjusted to the mechanical cutting plane in **(B**_**1**_**)**. **(B**_**2**_**)** is a detail of **(B**_**1**_**)**. The semithin section in **(B**_**2**_**)** especially shows that the swim bladder extension is firmly attached via connective material to a thin bony lamella which directly borders on the lagena of the inner ear. The white arrowhead in **(B)** indicates a disruption of the swim bladder wall; an artifact not observed in the tomographic section (white arrowhead in **(A)**). In contrast to the tomographic section, only a small part of the otolith is left due to decalcification in the histological sections. For the section plane refer to Figure 1. Scale bars, 1 mm **(A)**, 500 μm **(B**_**1**_**)**, 250 μm **(B**_**2**_**)**. microCT, microtomography; SL, standard length.

**Figure 3:**
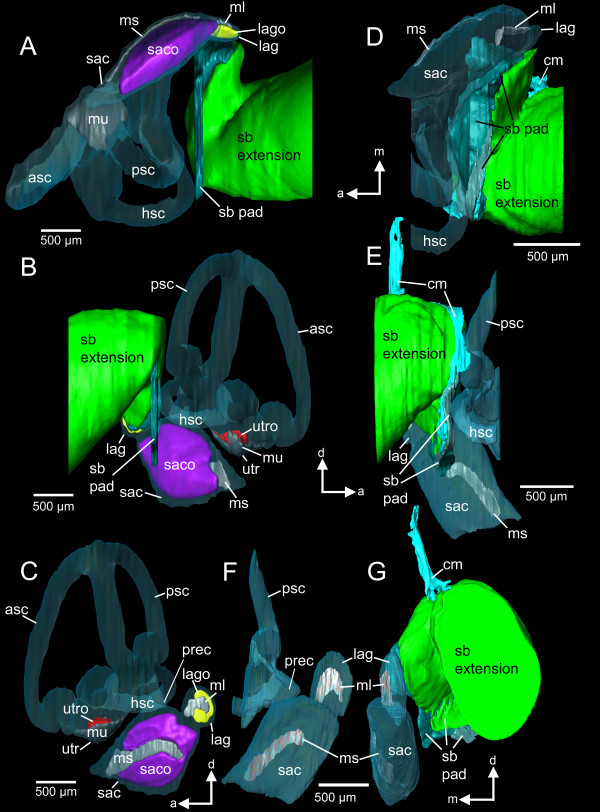
**Three-dimensional reconstruction of the right inner ear and swim bladder extension of *E. maculatus*.** Reconstructions are based on high-resolution (voxel size 9.8 μm isotropic) microCT imaging **(A-C)** and histological serial sections (voxel size x = y = 2.31 μm; z = 5 μm) **(D-G)** shown in ventral **(A, D)**, right lateral **(B, E)**, medial **(C, F)**, and postero-lateral **(G)** views. In **(G)**, semicircular canals are not shown for reasons of clarity. a, anterior; asc, anterior semicircular canal; cm, connective material; d, dorsal; hsc, horizontal semicircular canal; lag, lagena; lago, lagenar otolith; m, medial; ml, macula lagenae; ms, macula sacculi; mu, macula utriculi; prec, recessus situated posterior to the utricle; psc, posterior semicircular canal; sac, saccule; saco, saccular otolith; sb, swim bladder; utr, utricle; utro, utricular otolith. Scale bars, 500 μm. microCT, microtomography.

The swim bladder extensions in *E. maculatus* (for an overview see Figure 1) showed a bipartite structure composed of a gas-filled portion and a pad-like part. The former contacted the lagena via connective material attached to a thin bony lamella (minimum 3 μm) bordering this end organ (Figure 2). The swim bladder pad almost contacted the posterior and horizontal semicircular canals and a recessus located posterior to the utricle (Figure 3).

**Figure 4:**
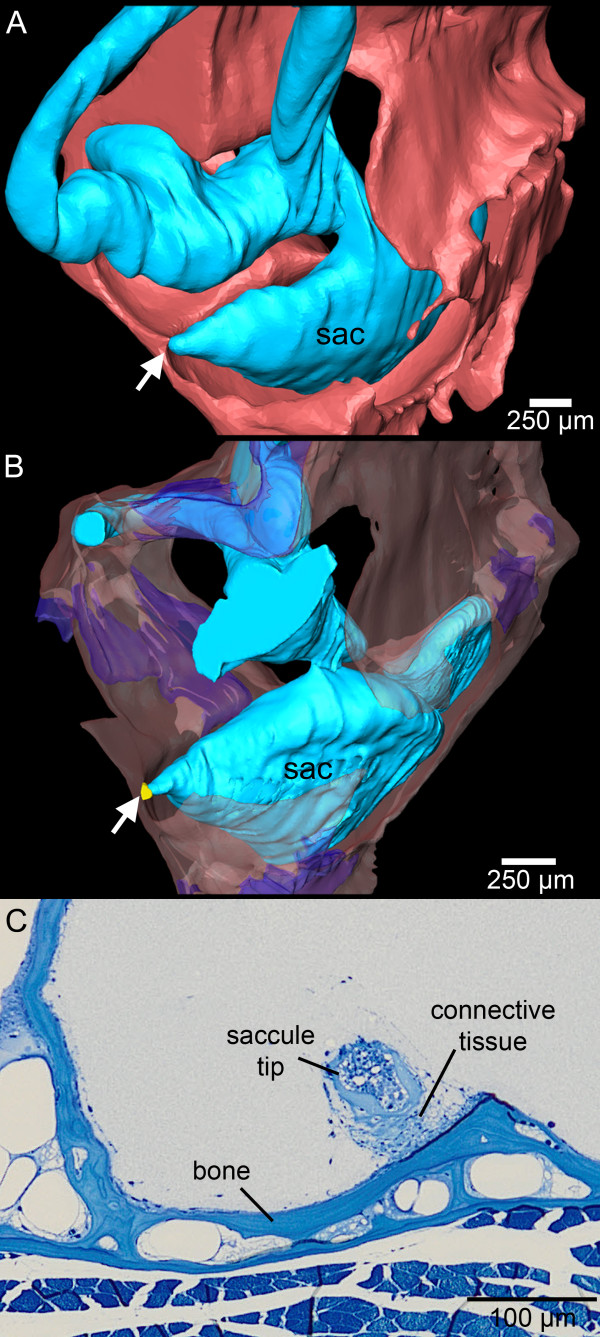
**The tip of the saccule in *E. maculatus *is firmly attached to the bone via connective tissue (shown in yellow, B).** Three-dimensional reconstructions are based on microCT imaging in **(A)** and histological serial sections in **(B)**. In **(C)** the connection is shown in a histological semithin section. sac, saccule. The white arrow in **(A)** and **(B)** indicates the attachment site. In **(A)** cranial bone and cartilage are both shown in red while in **(B)** bone is transparent red and cartilage transparent dark blue. Scale bars, 250 μm **(A-B)**, 100 μm **(C)**. microCT, microtomography.

The saccule did not show a link to the swim bladder but its anterior tip was firmly attached via connective tissue to the medial cranial bony wall surrounding the saccule (Figure 4).

### Swim bladder-neurocranium attachment

**Figure 5:**
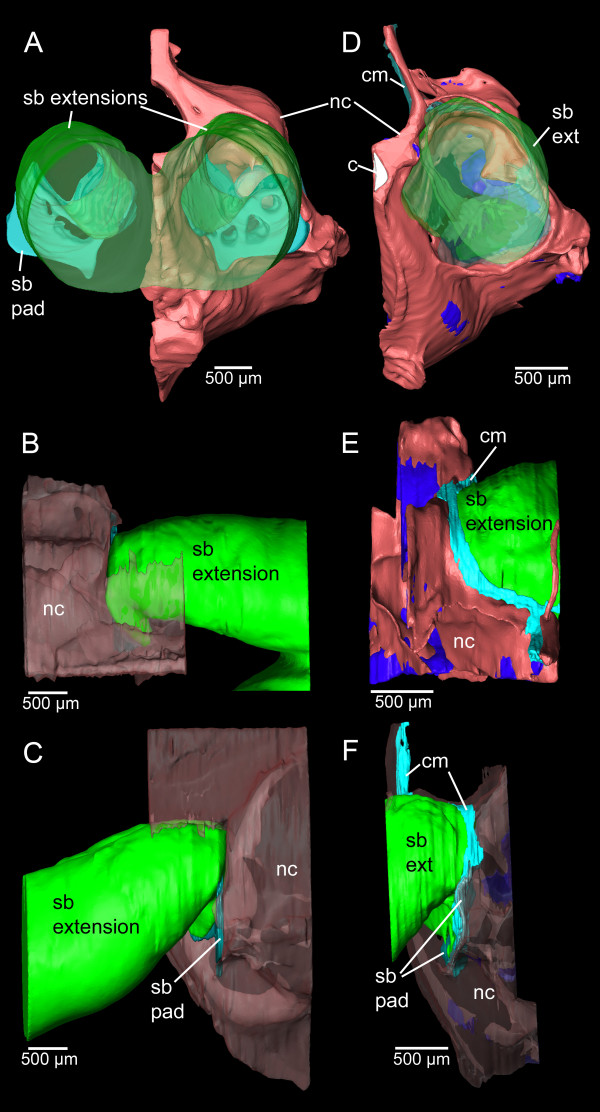
**Three-dimensional reconstruction of neurocranium and swim bladder extensions of *E. maculatus*.** Reconstructions are based on microCT imaging **(A-C)** and histological serial sections **(D-F)** shown in posterior **(A, D)**, dorsal **(B, E)**, and right lateral **(C, F)** views. c, chorda (white); cm, connective material (light blue); nc, neurocranium (red, transparent red, or red (bone) and dark blue (cartilage); sb pad, swim bladder pad (blue); sb ext, swim bladder extension (green). Scale bars, 500 μm. microCT, microtomography.

No internal bullae were identified within the neurocranium. The gas-filled part did not penetrate the skull but was connected to bone surrounding the lagena (see description above). The swim bladder pad closes the large exoccipital foramen. The gap between neurocranium and extension was sealed by a ribbon of connective material stretching across the dorsal and lateral to the ventral sides of the swim bladder extension (Figure 5).

### Structure of the swim bladder pad

**Figure 6:**
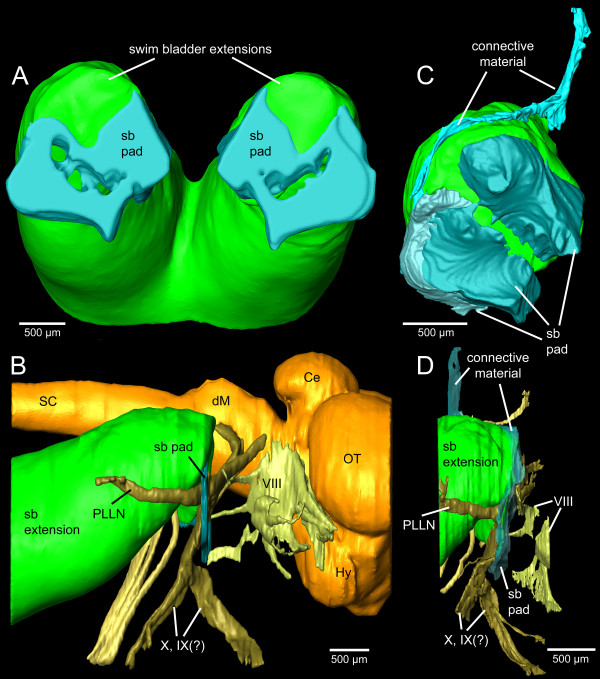
**Three-dimensional reconstruction of swim bladder extensions, brain, and nerves of *E. maculatus*.** Reconstructions are based on microCT imaging **(A-B)** and histological serial sections **(C-D)** shown in anterior **(A, C)** and right lateral **(B, D)** views. **(C)** and **(D)** show the right swim bladder extension only. Ce, cerebellum; dM, dorsal medulla; Hy, hypothalamus; OT, optic tectum; PLLN, posterior lateral line nerve; sb pad, swim bladder pad; SC, spinal cord; VIII, octaval nerve; (?)IX, glossopharyngeal nerve; (?)X, vagal nerve. Scale bars, 500 μm. microCT, microtomography.

**Figure 7:**
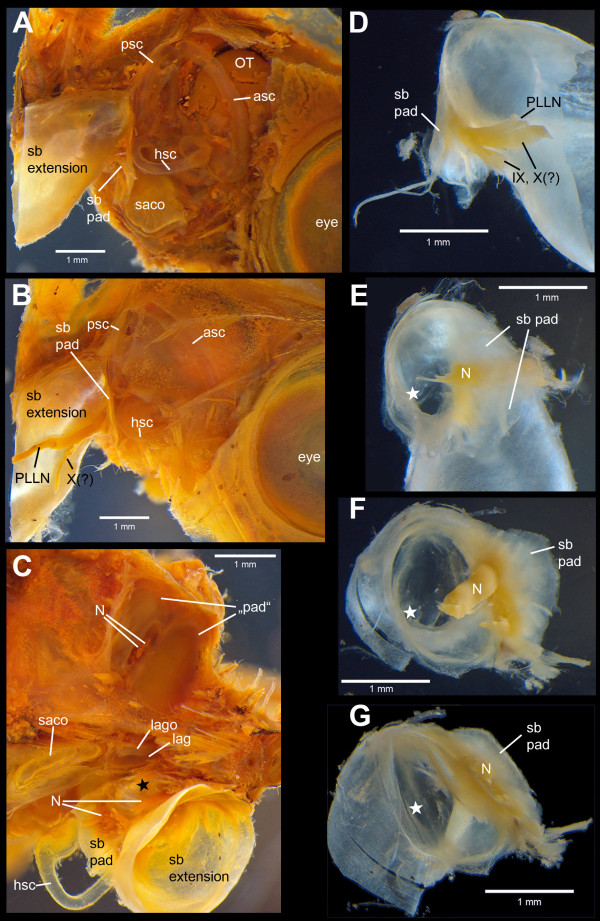
**Iodine stained specimens of *E. maculatus *with swim bladder extensions and inner ears exposed.** Lateral views **(A-B)** show the close vicinity of the swim bladder pad and the posterior and horizontal semicircular canals. In the ventral view **(C)**, the swim bladder extension of the right side indicates the contact between the gas-filled part of the extension and the lagena. On the left side, the extension was detached exposing a ‘tissue sheath’ (‘pad’) closing the exoccipital foramen, the former only penetrated by nerves. Detached swim bladder extensions **(D-G)** display the gas-filled portion and parts of the pad shown in lateral **(D)**, anterior **(E)**, antero-medial **(F)** and ventro-medial **(G)** views. Black **(C)** and white **(D-G)** asterisks indicate the gas-filled part of the anterior most swim bladder extension contacting the lagena. asc, anterior semicircular canal; hsc, horizontal semicircular canal; lag, lagena; lago, lagenar otolith; N, nerves (PLLN, X, and/or IX); OT, optic tectum; PLLN, posterior lateral line nerve; psc, posterior semicircular canal; sb pad, swim bladder pad; saco, saccular otolith; (?)IX, glossopharyngeal nerve; (?)X, vagal nerve. Scale bars, 1 mm.

**Figure 8:**
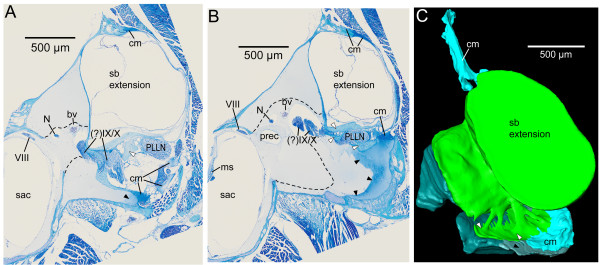
**Structure of the swim bladder pad in *E. maculatus *shown in transverse semithin sections (A-B) and as a three-dimensional reconstruction (C).** The swim bladder pad is composed of four components: (1) parts of the swim bladder wall (white arrowheads), (2) connective material (cm, light blue) dorsally and laterally attaching the swim bladder extension to the skull, (3) connective material in the ventral portion of the pad (black arrowheads), and (4) connective material (limited by dashed lines and shown in dark green) in the ventro-medial part of the pad. bv, blood vessel; cm, connective material; N, nerve; ms, macula sacculi; PLLN, posterior lateral line nerve; prec, recessus situated posterior to the utricle; sac, saccule; VIII, ocataval nerve; (?)IX/X, glossopharyngeal and vagal nerves. Scale bars, 500 μm.

The swim bladder pad was penetrated by a large blood vessel and the glossopharyngeal, vagal, and posterior lateral line nerves (Figures 6, 7). Four pad components were identified: (1) parts of the swim bladder wall that made up the postero-ventral region; (2) connective material connecting the extension to the exoccipital foramen, reaching from the supraoccipital crest to the ventral portion of the pad; (3) connective material contacting the lagena as well as coming close to the horizontal and posterior semicircular canals; (4) connective material building up the ventro-lateral part (Figure 8). The connective materials seem to have mainly a gelatinous character.

### Methodological comparison

The microCT and histological imaging methods complemented each other to produce a more complete and detailed picture of the anatomical structures and relationships in the inner ear. Certain details are more visible in histological preparations, while more of the three-dimensional relationships among structures are better demonstrated with microCT.

The reconstruction of otoliths was based on microCT imaging (Figure 3A-C versus D-G: without otoliths). Organic remains of otoliths after decalcification for the histological analysis did not allow reliable labeling: the remains were insufficient to reconstruct the otolith contour or were detached from the sensory epithelium through decalcification, dehydration, and embedding procedures (Figure 2B).

Components of the swim bladder pad could be identified in their full extent based on the histological serial sections (Figure 8), whereas only part of the pad (regions of best contrast between pad and other tissues) could be traced using microCT imaging (Figure 3A versus D). Similarly, differentiation between bone and cartilage was only possible based on histological serial sections (Figure 5E versus B). Sensory epithelia (maculae) could be identified on tomographic sections; however, reconstruction was especially difficult for the regions of the maculae not covered by the otoliths (compare Figure 3C versus F). Results of all three-dimensional reconstructed structures based on both methods can interactively be viewed and compared in Additional file 1.

## Discussion

For the first time, we describe in detail a link between the swim bladder and the lagena in a teleostean fish. Moreover, the swim bladder extension showed a unique, complex bipartite structure that also approaches the recessus located posterior to the utricle. This may have implications for the stimulation pathways from the swim bladder to the inner ears.

### The swim bladder-lagena connection

In most fish with accessory hearing structures and improved auditory abilities, the swim bladder or other gas-filled cavity comes close to or is connected to the saccule. Examples for such a morphological specialization are found in some species of the Holocentridae (*Myripristis*), Gerreidae (*Eucinostomus argenteus*), Notopteridae, Mormyridae, Osphronemidae, and all otophysans [21-27]. This intimate relationship between swim bladder/gas bubble and saccule is often correlated with higher auditory sensitivities and/or expanded hearing bandwidths [23,25,27]; for an overview see also [8]. It is, thus, reasonable to assume that the intimate contact between swim bladder extensions and lagenae in *E. maculatus* helps explain the improved auditory sensitivities.

### Potential function of the swim bladder pad

The pad may tightly attach the swim bladder extension to the neurocranium and bring the extension close to the inner ear. The large area of the pad closing the exoccipital foramen may result in a large area of transmission of the swim bladder oscillation in a sound field to the inner ear. The vibrating pad may thus act on the whole perilymph surrounding the ear, stimulating the end organs in an unspecific manner. Parmentier *et al*. [25] hypothesized that the swim bladder horns in *Eucinostomus argenteus*, which contact the saccule only indirectly via the perilymph, may account for enhanced hearing abilities.

In the region of the recessus of the upper ear, the stimulation may be more specific because the gap separating pad and recessus is very small. Future studies should investigate the ultrastructure of the pad components to elucidate their mechanical properties and clarify the extent to which the pad efficiently transmits swim bladder vibrations to the inner ear.

### Effects of the saccule-to-bone attachment

The attachment of the anterior saccule tip to the bone represents a specialization: this condition was not found in other cichlids lacking swim bladder-inner ear connections, including *Hemichromis guttatus*, *Steatocranus tinanti* (personal observation) or *Sarotherodon melanotheron melanotheron*[28]. At least two species with swim bladder-saccule connections via a bony lamella, namely *Antimora rostrata* (Moridae) and *Chitala chitala* (Notopteridae), possess a similar attachment of the saccule to the cranial bone [24,29,30]. Deng *et al*. [30] proposed that this mechanical link between swim bladder and saccule, both attached to the cranial bone, may directly stimulate the end organ through sound pressure fluctuations of the swim bladder transmitted by the bone to the saccule. A similar stimulation pathway may be suggested for the attached saccule of *E. maculatus*, although the swim bladder extension is closer to the bony wall of the lagena than to the saccule. This would imply a transmission of the vibration from the bony wall at the lagena along the bone to the saccular tip. Unfortunately, no information is available about the auditory capacities of *A. rostrata*. In *C. chilata,* displaying a similar swim bladder-saccule-bone relationship, auditory sensitivities and hearing bandwidth were comparable to species lacking accessory auditory structures [24,29]. This species did have better frequency selectivity compared to species lacking swim bladder-inner ear connections (*Osteoglossum bicirrhosum*, Osteoglossidae; *Sargocentron xantherythrum*, Holocentridae) [24,29]. Moreover, in otophysans, such as the goldfish, mechanical coupling of the swim bladder with the inner ears via a chain of ossicles (Weberian ossicles) results in high auditory sensitivities and distinctly expanded hearing bandwidths [20,21,31-33].

On the one hand, the morphological specializations in *E. maculatus* may function to increase auditory sensitivities and expand hearing bandwidth, as shown in previous studies [15,34]. On the other hand, they could also serve to improve other aspects of hearing, such as frequency selectivity (see the above mentioned *Chilata* example), directional hearing or the vestibular sense and, thus, maneuverability of fish. The bipartite structure of the swim bladder extension in *E. maculatus* contacting parts of the upper and the lower inner ear, may therefore point to multifunctional refinements.

Loricarioid catfishes and clupeiformes are other examples which evolved swim bladders that are simultaneously linked to two parts, in this case to the inner ears and the cephalic lateral line system [35,36]. Yet, the function of the proximity of the swim bladder to the lateral line system (laterophysic connection) in these catfishes is unknown. The link between intracranial gas bubbles of the swim bladder to the utricle in clupeids [37-39] was believed to enable some species to detect ultrasound [37,38,40]. A more recent study provides evidence that improved hearing abilities, that is, ultrasound detection, may at least partly reflect the laterophysic connection in these species [41].

### Methodological considerations

Three-dimensional reconstruction based on histological serial sectioning and high-resolution microCT imaging is suitable only for relatively small samples. Nonetheless, the combination of the two techniques successfully compensates for artifacts, such as disruption (for example, Figure 2B_1_ versus A) and incomplete hard structure visualization after decalcification during histological analyses, and the paucity of reliable tissue discrimination in microCT imaging [42]. Our study shows that the approach is even more powerful if non-destructive and destructive methods are combined within one specimen. This is because structural variability can definitively be assigned to embedding and sectioning procedures and recognized as artificial. The exact effects of initial fixation, however, cannot be identified as all these methods use already fixed samples. Applying CT imaging to live fish is possible [43] but still lacks the high resolutions necessary for reconstructing and interpreting the fine structures investigated here. Finally, the workflow employed here has the additional advantage of enabling morphological and histological analyses of specimens whose individual auditory sensitivities were previously measured.

## Conclusions

**Figure 9:**
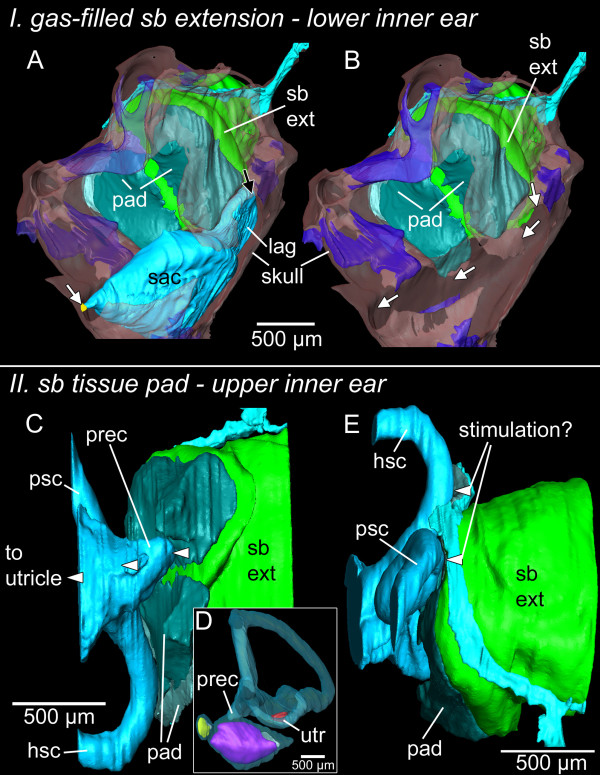
**Hypothetical model of inner ear stimulation by the bipartite swim bladder extension in *E. maculatus*.** I, lower inner ear: the gas-filled part of the swim bladder extension (green) may stimulate the lagena (light blue) via a thin bony lamella (transparent red) **(A**, black arrow**)** and the transmitted pressure signal may be further transduced via bone through the saccule (light blue) attachment (yellow) to the saccule **(A** and **B**, white arrows**)**. II, upper inner ear: the pad (dark green and silver) may transduce vibration to the recessus (light blue) and may thus indirectly stimulate the utricle **(C-D)**. It is noteworthy that the pad also comes very close to the posterior and horizontal semicircular canals (light blue) **(E)**. Bone is shown in transparent red, cartilage in transparent dark blue. hsc, horizontal semicircular canal; lag, lagena; psc, posterior semicircular canal; prec, recessus situated posterior to the utricle; sac, saccule; sb ext, swim bladder extension; utr, utricle. Scale bars, 500 μm.

The peculiar structure of the swim bladder extensions in *E. maculatus* (contacting the upper and lower parts of the inner ear, along with the saccule attachment discussed above) points to an involvement of all three end organs in improved auditory abilities (Figure 9). Sound pressure may be transduced from the swim bladder extension to the lagena via the thin bony lamella (Figure 9A) and may further lead to a vibration of the medial bony wall flanking the saccule. This would transmit the signal to the link between the bone and the saccular tip (Figure 9B). Because the most intimate contact exists between the gas-filled part of the swim bladder extension and the lagena, we suggest that the lagena partly accounts for the improved auditory abilities in this species.

Oscillation of the swim bladder may also be mediated by the pad to the nearby recessus and, thus, to the utricle (Figure 9C-D). We hypothesize that the saccule and utricle, therefore, receive indirect input from the swim bladder as well. It remains elusive whether the pad has any effect on the semicircular canals (Figure 9E).

Our study additionally demonstrates that a combined application of high-resolution microCT and histological serial sectioning on the same sample is a powerful method. It yields simultaneous, detailed three-dimensional insights into the microanatomy of hard parts and soft tissue compensating for weaknesses of each separate method. Moreover, this approach can be applied to a wide range of small biological samples and may, thus, be of interest for morphological studies in general.

## Methods

*E. maculatus* originated from local fish suppliers and were transferred to the University of Vienna in August/September 2011 and January 2012 (large individuals for dissections) and May 2012 (small individuals for microCT, histological analyses, and hearing measurements). Animals were kept in 98-L and 245-L aquaria, which were equipped with a sand bottom, halved flower pots as hiding places and external filters. Fish were kept under a 12:12 hour light:dark cycle at 25°C and were fed once daily with commercial flake food and red blood worms.

### Auditory measurements

As rather small individuals were used to ensure high resolution for microCT imaging and to facilitate thin sectioning for histology, we measured auditory sensitivities for control purposes and compared the thresholds gained with data on larger *E. maculatu*s (mean SL = 37 mm) measured in the prior study by Schulz-Mirbach *et al*. [15]. Hearing thresholds were measured in four individuals (mean SL = 28 mm) at 0.5, 1, 2, and 3 kHz using the AEP recording technique. We chose these frequencies because it was previously shown that *E. maculatus* has significantly higher auditory sensitivities than other cichlid species at frequencies ≥0.5 kHz [15]. The experimental set-up and methodological procedure followed exactly those described in Schulz-Mirbach *et al*. [15]. All auditory experiments were performed with the permission of the Austrian Federal Ministry of Science and Research (permit number GZ 66.006/0023-II/10b/2008).

### X-ray microtomography

First, three out of the four audiometrically measured specimens (Table 2) were anesthetized and euthanized using a concentration of about 0.2 mg/ml of MS 222 (Sigma-Aldrich Inc., Vienna, Austria), fixed in 10% buffered formalin at 4°C for two days and then stained in a near-isotonic Lugol solution (2.5% potassium iodide (KI) and 1.25% elementary iodine (I_2_) in water [44]) at room temperature for one week in order to enhance tissue contrast.

**Table 2:** **Number of specimens, standard length (SL) and methods used for investigating the connection between swim bladder and inner ears in *E. maculatus***

**SL (mm)**	**microCT scans (overview)**	**microCT (high-resolution)**	**Histology**	**Dissections**
25, 28, 29	3			
29		1	1	
33, 41, 45				3

*microCT* microtomography.

Overview scans of whole fish (Figure 1) were performed using a SkyScan 1174 microCT system employing a 50 keV/40 W tungsten X-ray source; a 1.3 megapixel CCD camera was used. The images were scanned using isotropic resolution (voxel size 36 μm isotropic), were reconstructed without binning and were finally stored as BMP image stacks. A ring-artifact-reduction utility was engaged (setting 7 to 10) during reconstruction for all the images. Labeling and three-dimensional reconstruction of otoliths and swim bladder revealed that all specimens showed the same close relationship of otoliths (as parts of the inner ears) and the swim bladder with its extensions (Figure 1). Subsequently, one of the three individuals was chosen for further detailed investigations.

High-resolution scans of the inner ear and the anterior most part of the swim bladder of one specimen were performed with a MicroXCT high-resolution microCT system from Xradia Inc., Pleasanton, CA, USA [45] with a tungsten X-ray source and variable secondary optical magnification. These scans were made with an anode voltage setting of 80 kV at 8 W and using the 2X objective with an exposure time of 30 seconds for projection images every 0.25°. Image stacks (voxel size 9.8 μm isotropic) were then edited in AdobePhotoshop® CS2, reduced from 16 bit to 8 bit grayscale and cropped.

### Histology

Following the high-resolution scans, the head was removed and cut into two halves. The right side was decalcified using a 2% solution of ascorbic acid for three days at room temperature. The brain was removed followed by subsequent dehydration through an ascending acetone series (30, 50, 70, 90, 95, 99, and 4 steps at 100%). The sample was then embedded in Epon (Carl Roth GmbH & Co. KG, Karlsruhe, Germany). Light microscopical and histological procedures followed Ruthensteiner [46]. Serial sectioning (1 μm) was performed with a ‘Histo Jumbo’ diamond knife (Diatome AG, Biel, Switzerland) on a MT XL ultramicrotome. Semithin-sections were stained with Richardson’s solution [47].

Images of whole slides were taken using an Olympus BX61VS microscope (objective: 10X; Olympus, Hamburg, Germany) equipped with a CX10 digital camera and a dotSlide system to circumvent manual post-stitching. Using the software OlyVia 2.4 (Olympus Soft Imaging Solutions GmbH), snapshots of every fifth section were digitalized with a voxel size of x = y = 1.82 μm, z = 5 μm. In AdobePhotoshop® CS2 images were converted from RGB to grayscale, cropped and reduced to a final voxel size of x = y = 2.31 μm, z = 5 μm.

### Three-dimensional reconstructions

Three-dimensional renderings of inner ears (including otoliths and sensory epithelia), brain (based only on microCT scans), nerves, swim bladder extensions and parts of the neurocranium were made in AMIRA® v. 5.4.0 (Visage Imaging GmbH, Berlin, Germany). For labeling structures in the microCT generated stack, mainly a threshold-based segmentation was applied; if necessary, this labeling was refined or corrected using the brush tool. For the image stack based on the histological serial sectioning, manual labeling was performed by using the brush tool only. For reconstructing the otoliths and organs, initially every third to fifth image was labeled, with subsequent interpolation of structures on intervening images, followed by a manual check and correction of segmentation results if required.

Subsequently, every otolith and organ was separated from the ‘master’ LabelField file into single LabelFields and saved as separate files. Surface rendering was performed with the SurfaceGen module. If necessary, surfaces of each labeled object were reduced to 100,000 surfaces. This was followed by smoothing the surfaces using the SmoothSurface module (20 iterations; unconstrained smoothing).

In order to compare the success of both methods, we illustrated three-dimensional reconstructions of structures based on microCT imaging as well as on histological serial sectioning. Moreover, in Figure 2 we compared a single tomographical section using the ‘ObliqueSlice-Tool’ in AMIRA with histological sections. This was done to orient sections based on both methods (with slightly different angle of almost transversal planes) towards the same section plane.

### Dissections of iodine-stained samples

Three additional specimens (Table 2) were fixed in 10% buffered formalin and stained with Lugol solution at room temperature for three hours up to one day. Specimens were then dissected to expose the swim bladder, inner ears, and the neurocranium or the brain.

For each sample a stack of 20 to 40 images at different focal planes was taken using a Leica MZ 16 F stereomicroscope equipped with a ProgRes® C5 camera, applying the ProgRes® MAC CapturePro 2.7.6 image capture software (Jenoptik AG, Jena, Germany). To combine the in-focus areas of the source images within each stack to an extended-focus two-dimensioal image, we created montage images using the ‘Do stack’ tool in CombineZM (Image Stacking Software by Alan Hadley, UK).

## Abbreviations

AEP: Auditory evoked potential; microCT: Microtomography; SL: Standard length.

## Competing interests

The authors declare that they have no competing interests.

## Authors’ contributions

TSM, MH, BM and FL conceived the study. TSM carried out the preparationsc and analyses except microCT imaging (BM) and histological serial sectioning. MH and BM provided support with the three-dimensional reconstructions. MH created Additional file 1. TSM, MH, BM and FL contributed equally to the writing of the manuscript. All authors read and approved the final manuscript.

## Supplementary Material

Additional file 1 **Three-dimensional reconstruction showing the relationship of the neurocranium, swim bladder extension(s), and inner ear in *Etroplus maculatus *based on microCT imaging and histological serial sectioning (interactive three-dimensional PDF).** Click on the figure to activate the three-dimensional features. For creation of interactive three-dimensional pdfs see also Ruthensteiner and Heß [48]. Click here for file
