# Supplementary material for: A unique swim bladder-inner ear connection in a teleost fish revealed by a combined high-resolution microtomographic and three-dimensional histological study
Source: BMC Biol. 2013 Jul 4;11:75. doi: 10.1186/1741-7007-11-75 (PMC3720219; doi:10.1186/1741-7007-11-75)
Supplement: Additional file 1 — Three-dimensional reconstruction showing the relationship of the neurocranium, swim bladder extension(s), and inner ear in Etroplus maculatus based on microCT imaging and histological serial sectioning (interactive three-dimensional PDF). Click on the figure to activate the three-dimensional features. For creation of interactive three-dimensional pdfs see also Ruthensteiner and Heß [48]. [file 1741-7007-11-75-S1.pdf]

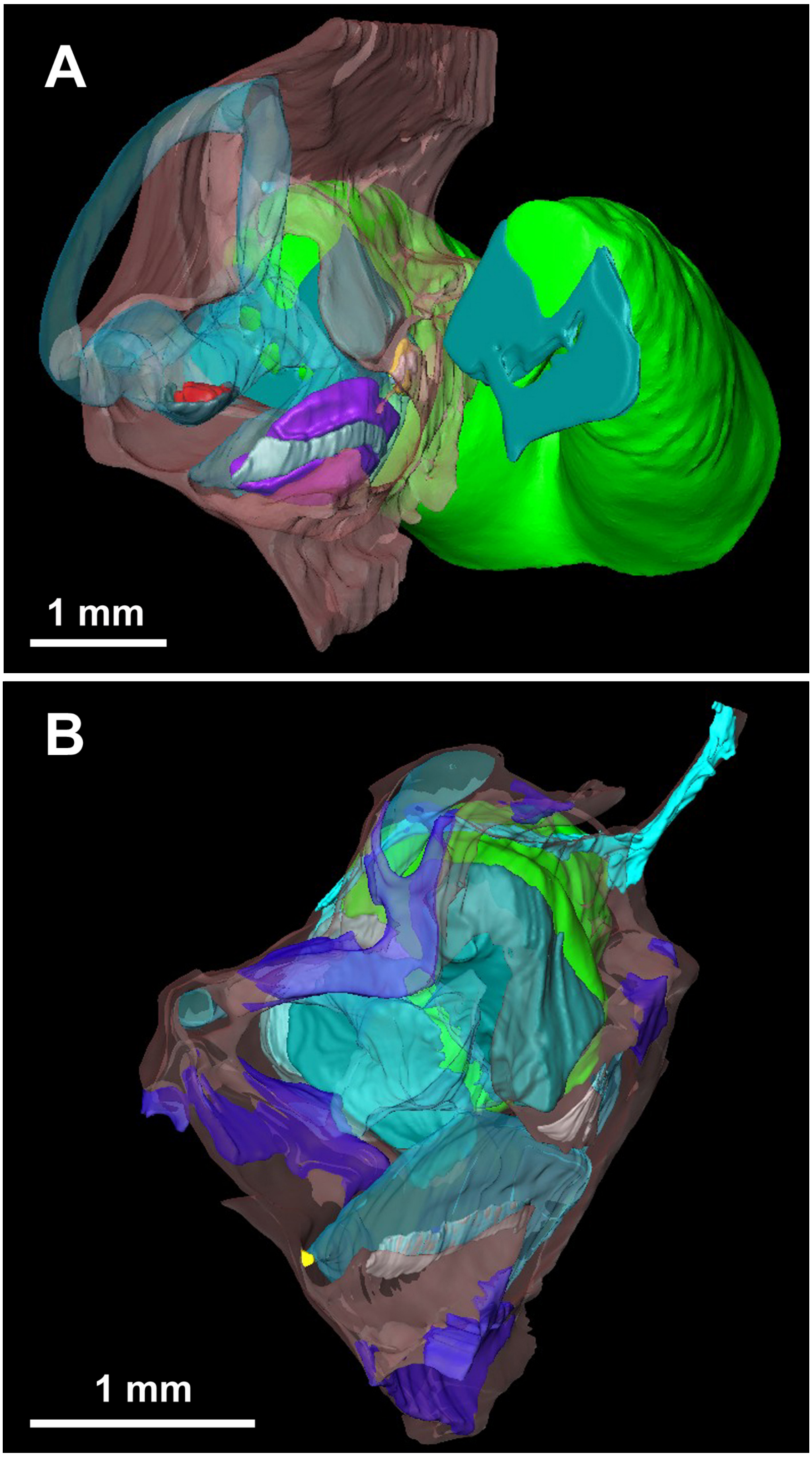

Supplementary Figure 1: Reconstruction of right inner ear and swim bladder extension of *Etroplus maculatus*. The **interactive 3D models** based on µCT (A) and semithin section series (B) can be accessed by clicking into the Figures (Adobe Reader Version 7 or higher required). Rotate model by dragging with left mouse button pressed, shift model: same action + ctrl, zoom: use mouse wheel (or change default action for left mouse button). Select or deselect (or change transparency of) components in the model tree, switch between prefab views or change surface visualization (e.g. lightning, render mode, crop etc.). Deactivate 3D content via context menu (right mouse clic).
